# Experimental Investigation of Shear Keys for Adjacent Precast Concrete Box Beam Bridges

**DOI:** 10.3390/ma15041459

**Published:** 2022-02-16

**Authors:** Xiaojing Ni, Ahehehinnou Ougbe Anselme, Guannan Wang, Yuan Xing, Rongqiao Xu

**Affiliations:** 1 Department of Civil Engineering, Zhejiang University, Hangzhou 310058, China; nxj54837@126.com (X.N.); aheanselme@hotmail.com (A.O.A.); guannanwang@zju.edu.cn (G.W.); 2 Huahui Engineering Design Group Co., Ltd., Shaoxing 312000, China; xingyuan1393@163.com; 3 Center for Balance Architecture, Zhejiang University, Hangzhou 310007, China

**Keywords:** bridge, adjacent box beam, shear key, shear experiments, overlay

## Abstract

Longitudinal cracking in shear keys is one of the most frequently recurring problems in the adjacent precast concrete box beam bridges. The relative displacement across the shear key (RDSK) under loads has been used as a direct indicator for shear key cracking. Therefore, accurately simulating the interface between the shear key and beam or providing the correct relationship between shear transfer and RDSK is key to evaluating the damage of the shear key. In this study, the shear transfer properties of four types of composite specimens were studied by static displacement-controlled bi-shear (SDS), cyclic force-controlled bi-shear (CFS), and cyclic displacement-controlled bi-shear (CDS) tests. Two finite element models (FEMs) were established to calibrate and validate the interfacial material parameters. The results showed that adding reinforcement bars over the joints that connect the block and the overlay could improve the bearing capacity of the shear key. Formulae were proposed for the relation between shear force transfer and RDSK in engineering applications. The values of the interfacial material parameters used in the traction–separation model to simulate the interface between the shear key and beam were recommended.

## 1. Introduction

Adjacent precast concrete box beam bridges are widely used in short- to medium-span bridges. However, one of the most significant issues for this type of bridge is the longitudinal cracking of the shear keys. Generally, it is believed that cracked shear keys compromise the load transfer between beams [1,2]. In extreme cases, the load on a single beam exceeds its designed allowable load, leading to accidents [3]. However, field observation also found that the load transfer between beams could still be maintained for partially cracked shear keys [4,5,6]. Therefore, it is necessary to reasonably evaluate the damage and load transfer capacity of shear keys to predict the remaining service life of bridges and select appropriate maintenance and reinforcement strategies.

Some researchers conducted destructive tests on structures to evaluate the load transfer performance of cracked shear keys. Wang et al. [7] carried out a static load test on a structure composed of six beams connected by concrete shear keys. They found that at a load level of 70 kN, two shear keys cracked with relative displacement across the shear key (RDSK) of approximately 0.02 and 0.04 mm, respectively. As the load increased, the crack in the shear keys propagated and eventually failed at a load level of 140 kN, twice the cracking load. Yuan et al. [8,9] conducted four tests on two-beam structures connected by transverse post-tensioning (PT) and partially or fully cracked shear keys, which were cast with nonshrink grout. Over millions of cycles, the load levels increased from 80 to 400 kN, and the PT force dropped from 445 to 0 kN. The results showed that when the transverse PT force decreases from 445 to 45 kN, the load can still be transferred effectively, and the RDSK remains stable. Miller et al. [10] carried out three cyclic loading tests on four-beam structures connected by transverse tie rods and shear keys, cast with nonshrink grout. In the first two tests, there were initial cracks at shear keys in the middle caused by temperature, and in the third test, there were initial cracks near the beam end. During the cyclic loading, the cracks in the first two tests propagated, while the cracks in the third test did not. They found that the load was effectively transferred, and the load distribution changed by no more than 1% during all three tests. However, according to the test result of Leng et al. [11], it may be due to the position of the load and crack. Leng et al. tested an eight-beam structure connected only by concrete shear keys. They set different crack lengths on the first and the fourth shear keys to assess the influence of crack length and transverse position on load distribution. They found that the crack at the first shear key had a significant impact on the load distribution, but that at the fourth did not. These destructive tests indicate that the cracking of shear keys does not mean load transfer failure, and the ultimate bearing capacity may be much larger than the cracking load. However, destructive tests are unsuitable for bridges in service to evaluate residual capacity; the finite element method is more appropriate.

Since RDSK has been used as a direct indicator for shear key cracking [4,7,8,9,12,13,14], it is crucial to accurately simulate the interface of the beam and shear key in finite element models (FEMs) using solid elements. Three commonly used methods are the full bond [15,16,17], friction [13,18,19,20,21], and traction–separation model [13,21,22,23]. Full bond is suitable for the interface of concrete and grout materials with strong bonding ability, such as epoxy, MgNH4PO4, and UHPC, but not for commonly used nonshrink grout and concrete. Shear and flexural tests showed that the former is more prone to cracks in the concrete, while the latter is more prone to cracks in the interface [8,9,15,17,23,24,25,26,27,28,29]. Friction applies when the shear key has already cracked at the interface. The traction–separation model can be used for all of the above materials, uncracked and cracked. To sum up, the traction–separation model is more suitable for the interface of concrete and grout materials such as nonshrink grout and concrete. However, the material parameters used in this model were usually reversely determined by direct tensile or direct shear tests [17,20,29]. The problem is that the interface of the two materials in these tests was flat; factors such as joint configuration and cast direction, which affect the properties of concrete-like materials [30,31], were not considered. Murphy et al. [17] simulated the shear test of joint specimens by material parameters obtained from these tests, resulting in much larger cracking loads in the simulation than those in the experiment. Material parameters determined from tests on joint specimens have not been reported yet.

The grillage method is another common method to simulate adjacent box-beam bridges to analyze the load distribution between beams [32,33,34,35]. Two transverse connection types are usually used, namely shear transfer systems and shear–flexure transfer systems. For the shear transfer system, beams are simulated as longitudinal grillage members with transverse outriggers, and shear keys are represented by the pinned joints between the outriggers of adjacent beams. Cracks in shear keys are indicated by lowered vertical stiffness, resulting in larger RDSK [36,37]. For the shear–flexure transfer systems, the longitudinal properties of beams are simulated by longitudinal grillage members; the transverse properties of beams and shear keys are simulated by equivalent transverse grillage members. Cracks in shear keys are indicated by the lowered stiffness of the transverse grillage members [38]. The shear transfer system is recommended for bridges with partial depth shear keys [32]. Although there have been many studies on the shear performance of shear keys, the focus was on cracking loads and maximum shear loads [15,17,20,23,24,25,28,29]. There was little quantitative information about the effects of shear key cracking on vertical stiffness or the relation between shear transfer and RDSK.

## 2. Objectives

The main objectives of this study were to investigate the relationship between shear transfer and RDSK for transverse connection before and after shear key cracking and determine interfacial material parameters between beams and shear keys cast with concrete. To this end, the following studies were performed in this work:

- Static displacement-controlled bi-shear (SDS), cyclic force-controlled bi-shear (CFS), and cyclic displacement-controlled bi-shear (CDS) tests were conducted on four types of composite specimens to investigate the shear transfer performance;
- Based on the test results, curves and expressions for the relation between shear and RDSK of different types of transverse connections were proposed;
- Two FEMs were developed to calibrate and validate the interfacial material parameters.

## 3. Experimental Program

### 3.1. Configuration

**Figure 1:**
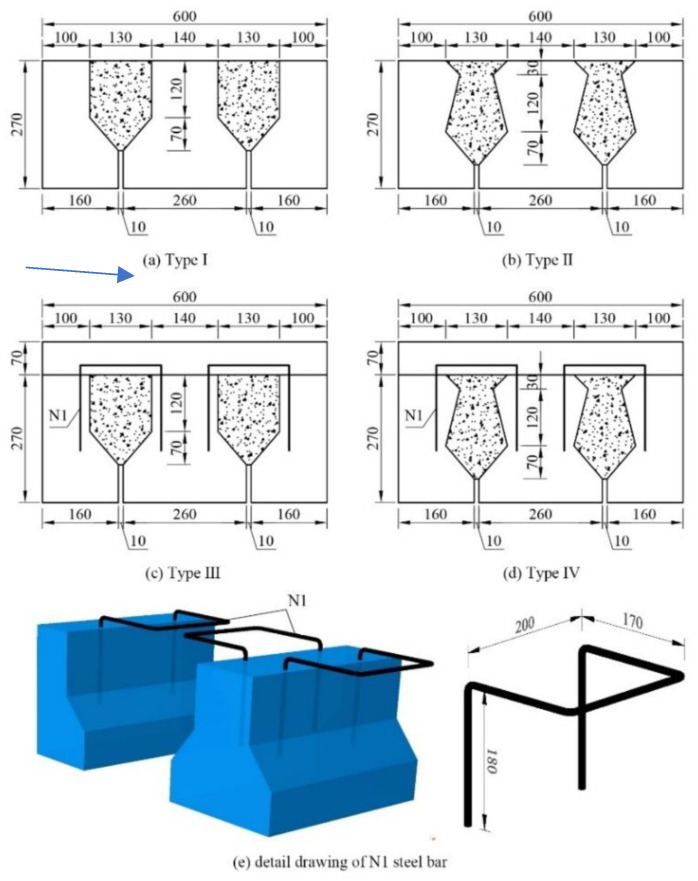
Configuration of specimens (note: all dimensions in mm): (**a**) Type I; (**b**) Type II; (**c**) Type III; (**d**) Type IV; (**e**) detail drawing of N1 steel bar.

Four connection details used to evaluate the shear transfer performance are shown in Figure 1. Type I and Type II specimens consisted of one concrete middle block and two concrete edge reaction blocks with grout joints between them. These two specimen types were 600 mm long, 270 mm in height, and 400 mm wide. Type III and Type IV specimens added a 70-mm-height concrete overlay and four 8-mm-diameter U-shaped steel bars (N1) based on Type I and Type II. Reinforcing steel bars in concrete blocks to prevent cracking are not drawn in Figure 1 for simplicity. Three-dimensional samples of N1 bars are shown in Figure 1e. N1 bars are spaced at 300–400 mm in the longitudinal direction in bridges; for safety reasons, 400 mm was used here as the width of specimens. Type II–IV connections had been widely used in Zhejiang Province in China before 2004 [39], and the bridges using these connection types now more or less experience longitudinal cracking problems. Type I was adopted here as a control for Type II and Type IV.

### 3.2. Specimen Preparation

**Figure 2:**
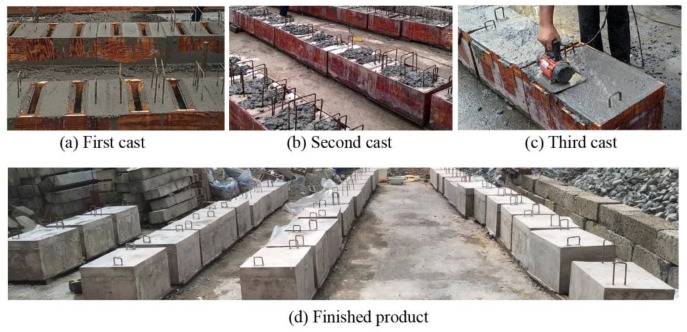
Specimen construction procedure.

The specimens were cast in three steps. Middle and edge concrete blocks were cast in the first step, joints in the second step, and overlays in the third step. During each cast, the concrete was vibrated by concrete vibrators to prevent imperfect filling. All steps were 30 days apart and moist-cured for 28 days. Companion cubes were cast synchronously at each step to determine the actual compressive strength of the cast material. The cohesion and friction properties of the interface between blocks and joints are mainly affected by the surface treatment of the blocks. In this study, the surfaces of blocks were roughened to an amplitude of 6 mm by a concrete scrabbler [40]. The whole procedure is presented in Figure 2. For better data acquisition by the digital image correlation (DIC) instrument, the front surfaces of specimens were polished, painted white, and black speckles added before tests.

Blocks and joints were constructed with commercial, ready-mixed concrete with a targeted 28-day compressive strength of 40 MPa (C40) and overlay of 30 MPa (C30) [41,42,43]. The constituents of the concrete are shown in Table 1. The average strength of the companion cubes was 46.1 and 45.2 MPa for C40 in the first and second cast and 34.6 MPa for C30 in the third cast [42]. N1 bars and stirrups in blocks had a nominal yield strength of 300 MPa (HPB 300) [44]. Other steel bars in blocks had a nominal yield strength of 400 MPa (HRB 400) [45].

### 3.3. Setup

**Figure 3:**
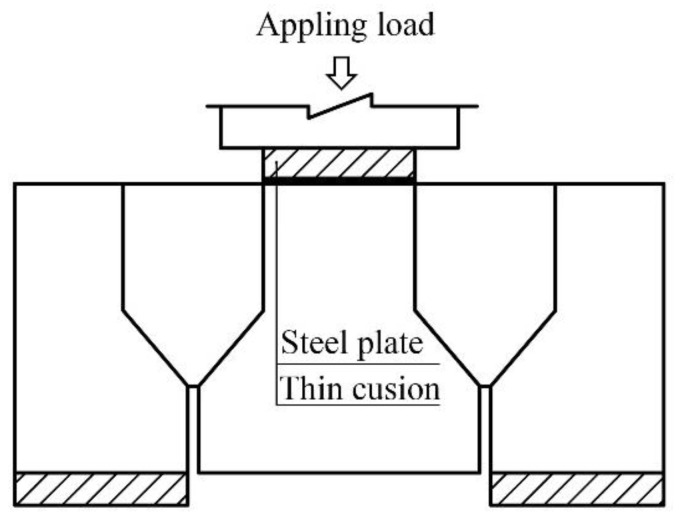
Test setup for bi-shear method.

**Figure 4:**
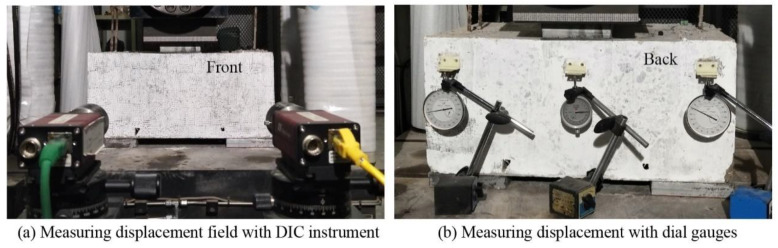
Layouts of instruments: (**a**) DIC instruments; (**b**) dial gauges.

**Figure 5:**
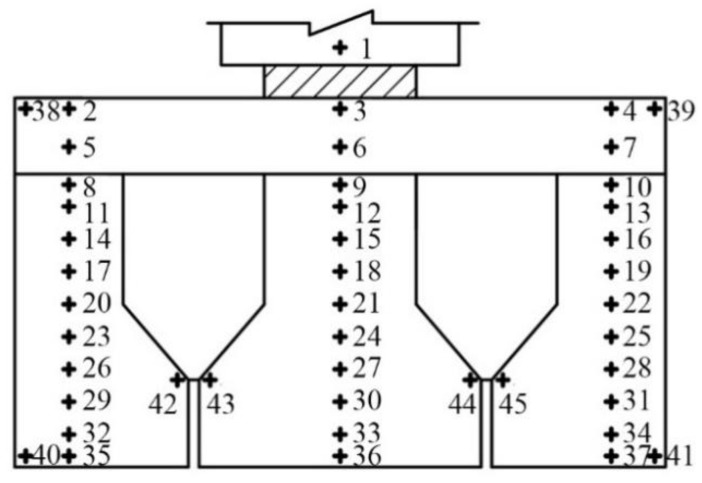
Data extracted positions.

The bi-shear test was performed using the setup depicted in Figure 3. A specimen was set on steel plates under edge blocks; the load was applied by a high-performance testing machine produced by INSTRON Company through a thin cushion and a steel plate placed on the top of the specimen. The machine could output displacement and load synchronously. The DIC instrument and dial gauges recorded displacement under load at the front and back surfaces, respectively (see Figure 4). DIC recorded the whole displacement field of the front surface during testing, and the data of specified points were extracted and analyzed for different purposes. Taking the front surface of the Type III specimen as an example (see Figure 5), Points 14 to 16 were used to calculate RDSK (Δ); Point 1 to compare with the displacement output by the loading machine; Points 2 to 37 to analyze displacement variation in the vertical direction; Points 38 to 41 to analyze boundary displacements, and Points 42 to 45 to calculate the cracking opening. On the back surface, dial gauges only recorded Points 14 to 16. Δ was calculated using Equation (1):

(1) $$\Delta ={\left(w_{14}+w_{16}\right)/2-w_{15}|}_{\mathrm{front}\;\mathrm{or}\;\mathrm{back}}$$

where *w*_i_ is the vertical displacement of Point i (i = 14–16).

### 3.4. Test Pocedure

Three different shear test types were conducted in sequence to evaluate the shear performance of four connection details before and after cracking, namely SDS, CFS, and CDS test.

In the SDS test, the displacement was applied at a rate of 0.02 mm/s until the specimen failed. The displacement field, cracks, associated cracking loads, and final failure mode were recorded during the test.

In the CFS test, the load varied linearly between 15 kN and the specified control force at a frequency of 1 Hz. In the first CFS test, the control force was set at approximately 60% of the cracking load obtained by the SDS test. This value was proposed on the assumption that the properties of the interface material are similar to concrete, for which 60% of the maximum tensile stress could be regarded as the elastic limit [46]. The control forces of the second and third CFS tests were determined based on the result of the last test. The controlled force increased if the specimen was uncracked in the previous test. Otherwise, it decreased. A total of 1400 cycles were applied in one test for two main reasons: (1) supposing that the structure was overloaded once a week over a 20-to-30-year period; and (2) too much data generated by DIC during the test, causing storage problems. An SDS test on the same specimen would follow if no cracks appeared after the CFS test. The displacement field and the failure mode were recorded during the test.

**Figure 6:**
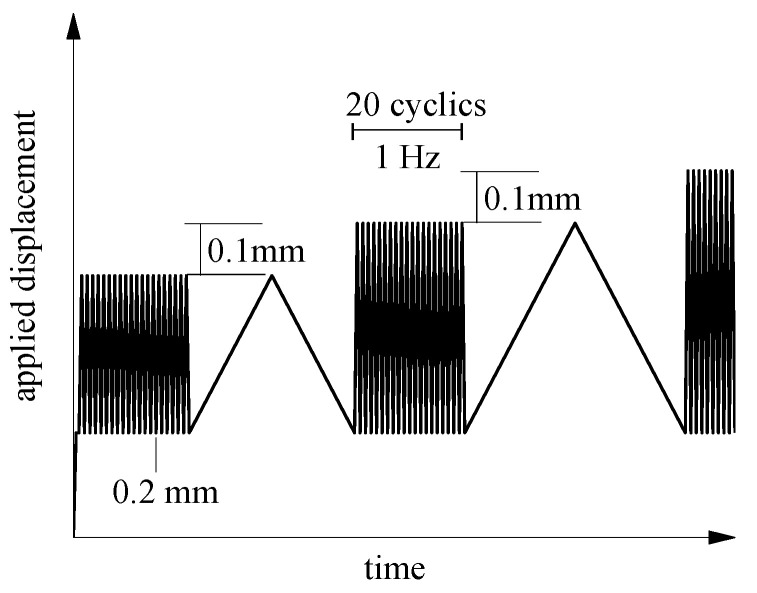
Loading procedure for CDS test.

In the CDS test, the displacement varied linearly between 0.2 mm and the specified control displacement at a frequency of 1 Hz. The CDS test was proposed based on the phenomenon that RDSK could remain steady after shear key cracking during cyclic loading [8,9]. In addition, to compare dynamic load capacity with static load capacity under the same displacement, one static loading was conducted every 20 cycles. The control displacement was increased by 0.1 mm after each static loading. Figure 6 shows the loading procedure. The displacement field and the failure mode were recorded during the test.

### 3.5. Results and Discussion

The following notation of specimen names was used throughout this study: the first three letters stand for the test type; the number following the first three letters represents the type of specimen, and the last digit indicates the replicate number of the specimen. For example, Specimen SDS-II-3 indicates the third Type II specimen subjected to the SDS test.

The following method was used to determine the average value: (1) the arithmetic mean value of the measured values was used as an average value, and (2) data greater than or less than 15% of the arithmetic mean were excluded from the mean calculation [41,42].

The value of shear force *V* was set to half of the load *F* output by the machine.

#### 3.5.1. Results of SDS Test

**Figure 7:**
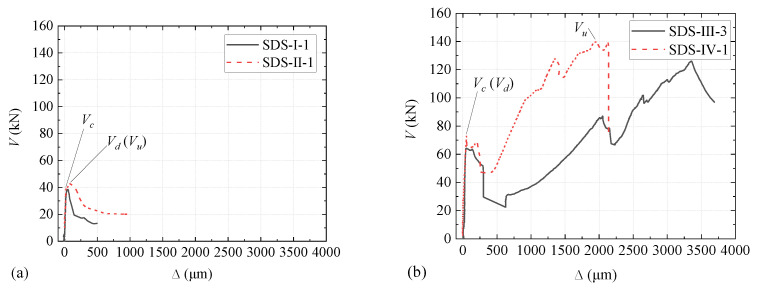
*V-*∆ plots of SDS test for typical specimens: (**a**) Specimen SDS-I-1 and SDS-II-1; (**b**) Specimen SDS-III-3 and SDS-IV-1.

**Figure 8:**
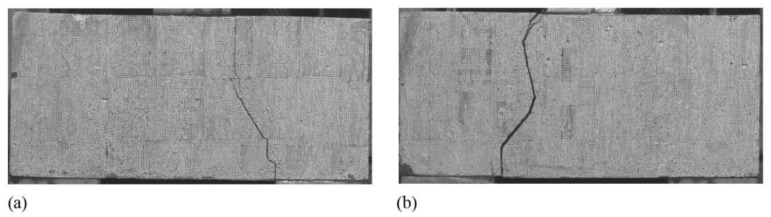
Failure modes of specimens: (**a**) Type I specimen; (**b**) Type II specimen.

**Figure 9:**
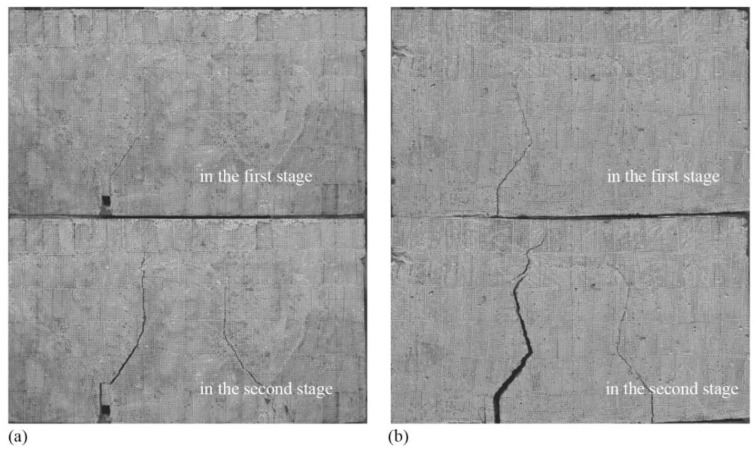
Failure modes of specimens: (**a**) Type III specimen; (**b**) Type IV specimen.

The typical *V*-∆ curves for four types of specimens are plotted in Figure 7. As shown, the *V*-∆ curves for Type I and Type II specimens are similar; the curves are approximately linear until a sudden failure occurs at the interface of block and joint. Figure 8 shows such a failure mode. The typical *V*-∆ curves for Type III and Type IV specimens are also similar, and the curves can be divided into two stages. In the first stage, the curves are similar to that of Type I and Type II; the interface of block and joint cracked during this stage, and the upper end of the crack extended to the bottom of the overlay (see Figure 9). In the second stage, *V* increases slowly and almost linearly with ∆ until another sudden failure. During this stage, the upper end of the crack slowly reached up to the top of the overlay (see Figure 9).

The cracking shear force (*V_c_*), the maximum shear force in the first ascending stage (*V_p_*), the minimum shear force in the second ascending stage (*V_d_*), the maximum shear force in the whole process (*V_u_*), the corresponding slips across the joint (∆*_p_* and ∆*_u_*), the stiffness before and after cracking (*k_c_* and *k_p_*), and crack opening (*C_o_*) for each specimen are listed in Table 2. The value of *k*_c_ is the slope of the ascending curve in the first stage, calculated by Equation (2):

(2) $$k_{c}=0.6V_{c}/\Delta_{0.6}$$

where ∆_0.6_ is the value of ∆ corresponding to 0.6*V_c_* on the *V-*∆ curve. The value of *k_p_* is the slope of the ascending curve in the second stage.

#### 3.5.2. Results of CFS Test

**Figure 10:**
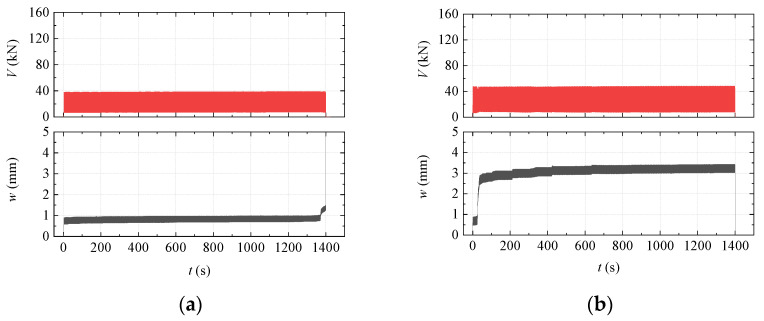
*V*-*t* and *w*-*t* plots of the CFS test for typical specimens: (**a**) Specimen CFS-II-2 and (**b**) Specimen CFS-IV-3.

The results of the CFS test are summarized in Table 3. The results agreed well with the previous assumption about the elastic limit (0.6 *V_c_*). Figure 10 shows specimens’ typical *V-t* and *w-t* curves during testing, where *w* is the displacement output by the machine and *t* is the time. *V-*∆ curves are not presented here because the specimen response lagged significantly behind the added load during cyclic loading.

For Type I and Type II specimens, once the specimen cracked, *w* increased quickly, and the specimen failed (see Figure 10a). In contrast, *w* found a new equilibrium position after several adjustment cycles for cracked Type III and Type IV specimens (see Figure 10b).

#### 3.5.3. Results of CDS Test

**Figure 11:**
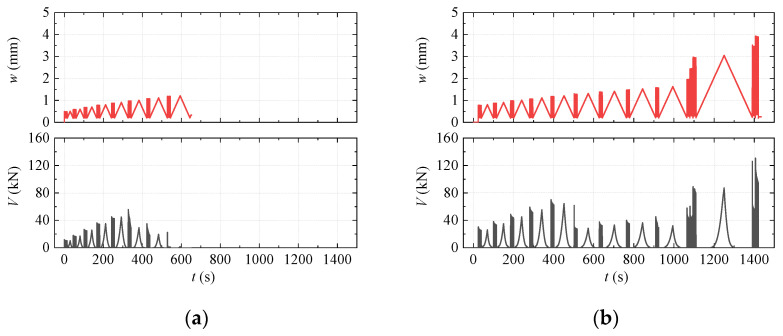
*w*-*t* and *V*-*t* plots of the CDS test for typical specimens: (**a**) Specimen CDS-II-1; (**b**) Specimen CDS-IV-2

The results of the CDS test are summarized in Table 4. The average cracking and maximum shear force under dynamic loading ($V_{c}^{d}$ and $V_{u}^{d}$) of Type I to Type IV did not change much compared to those of the SDS test. The typical *V-t* and *w-t* curves are shown in Figure 11. Once the specimen of Type I and Type II cracked, increasing *w* did not result in increasing *V* synchronously, and the specimen failed quickly (see Figure 11a). In contrast, *V* increased with *w* for the Type III and Type IV specimens after cracking and could remain steady when *w* was relatively low (see Figure 11b).

#### 3.5.4. Comparison of Different Connection Types

Using N1 bars and an overlay increased both the *V_c_* and *V_u_* of specimens. The *V_c_* and *V_u_* of the Type III connection were 1.5 and 3.4 times larger than those of the Type I connection, respectively. Moreover, the *V_c_* and *V_u_* of the Type IV connection were 1.2 and 2.9 times larger than those of the Type II connection, respectively. Type I and Type II connections failed quickly after cracking, while Type III and Type IV connections could still transfer shear force effectively. In addition, the shear transferring was steady when the force or displacement was not very high after cracking for Type III and Type IV connections.

#### 3.5.5. Relationship between *V* and ∆

Little research has been done on the relationship of *V* and ∆ for shear key connections used in adjacent box-beam bridges. Generally, when the shear key is intact, ∆ is assumed to be zero, and when the shear key is damaged, *V* is set to the product of ∆ and the stiffness of the shear key or just assumed as zero in the calculation [32,36,47].

**Figure 12:**
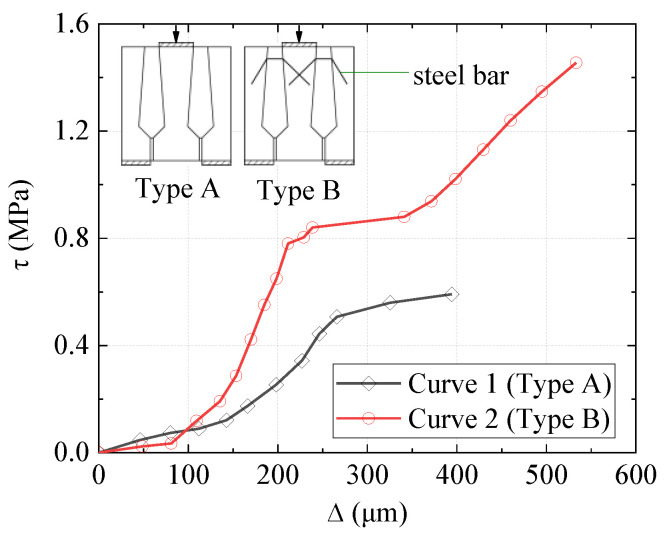
*τ-*∆ plots (data from Ye et al. [28]).

Ye et al. [28] investigated the shear performance of shear keys by monolithically increasing force-controlled bi-shear tests. They presented two typical τ-∆ curves, where τ was the shear stress obtained from dividing *V* by the interface area (see Figure 12). Both the blocks and joints were cast with concrete. Curve 1 and Curve 2 presented shear keys without and with reinforcing steel bars connecting blocks and joints, respectively. For both curves, stress τ increased with ∆ slowly before ∆ around 130 μm; then, an almost linear relationship between τ and ∆ was obtained until cracking. After the cracking, a minor increase in τ resulted in a large increase in ∆. The specimen without reinforcing steel bars failed at this time. Then, the specimen with reinforcing bars came into another almost linear relationship between τ and ∆ until it failed. Because the load was monolithically increasingly applied in their study, the load dropping after cracking for specimens with reinforcing bars was not captured. Uneven surfaces of specimens may explain why τ increased slowly with ∆ at the very beginning.

**Figure 13:**
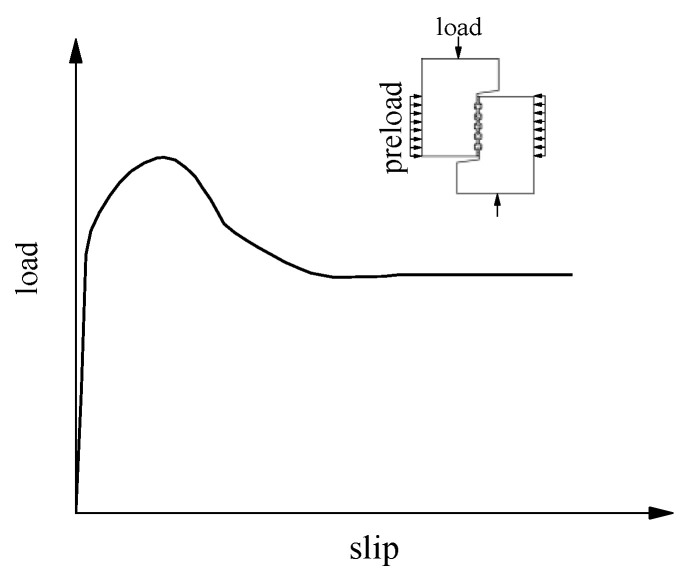
Load–slip curve (data from Rizkalla et al. [48]).

Rizkalla et al. [48] investigated the performance of flat and keyed joints used in shear wall panels with a compressive preload pressure of 2 MPa and 4 MPa normal to the shear-resistant surface under a direct shear test. Both outside blocks and the joint were cast with concrete. At first, the load was subjected to force control. After the maximum load was attained, the test continued with stroke control. The load–slip curves obtained from the test with different key configurations were similar. They presented a typical load–slip curve for multiple shear key connections, as shown in Figure 13. The curve is linear before cracking and has a load drop after the maximum load. For compressive stress used normal to the connection, the load remains steady even at large slips.

Instead of keyed joints, some researchers studied the relationship between the shear force and slip at two concrete interfaces cast at different times by bi-shear or direct shear tests [49,50,51]. The shapes of the shear–slip curves for specimens without and with steel bars at the interface are similar to those depicted in Figure 7a,b, respectively.

**Figure 14:**
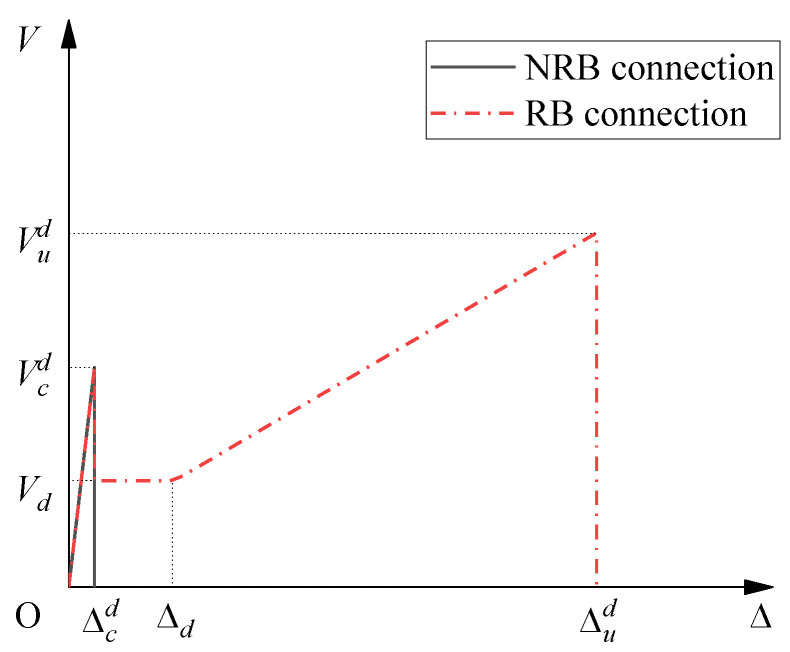
Typical *V*-∆ plots for NRB and RB connection.

Based on the results of all these tests, the *V*-∆ curves can be simplified into two types, one without reinforcing bars as Type I and Type II connection (NRB connection), and the other with reinforcing bars as Type III and Type IV connection (RB connection), as shown in Figure 14. As the results of the CFS and CDS tests showed that the elastic limit is around 0.6 *V_c_*, and when ∆ is in the descending part, the NRB connection could not provide steady shear transfer; the relationship between *V* and ∆ can be simplified as:

(3) $$V=\{\begin{array}{ll} k_{c}\Delta & \left(0\leq \Delta \leq \Delta_{c}^{d}\right)\\ 0 & \left(\Delta >\Delta_{c}^{d}\right) \end{array}$$

where 0.6 ∆_c_ is recommended for the value of $\Delta_{c}^{d}$. Similarly, the shear transfer in the second descending part for the RB connection can also be set to zero. In addition, the relationship between *V* and ∆ for the RB connection can be simplified as:

(4) $$V=\{\begin{array}{ll} k_{c}\Delta & \left(0\leq \Delta \leq \Delta_{c}^{d}\right)\\ V_{d} & \left(\Delta_{c}^{d}<\Delta <\Delta_{d}\right)\\ V_{d}+k_{p}\left(\Delta -\Delta_{d}\right) & \left(\Delta_{d}\leq \Delta \leq \Delta_{u}^{d}\right)\\ 0 & \left(\Delta >\Delta_{u}^{d}\right) \end{array}$$

where $\Delta_{u}^{d}$ is the correction value of ∆*_u_* based on the difference in static and dynamic motion.

## 4. FE Analysis

In the present study, FE analyses were performed using the software Abaqus 2018. Two FEMs were established, one based on the SDS test of the Type I specimen to calibrate the interface parameters and the other based on the Type III specimen to validate the interface parameters.

### 4.1. FEMs

**Figure 15:**
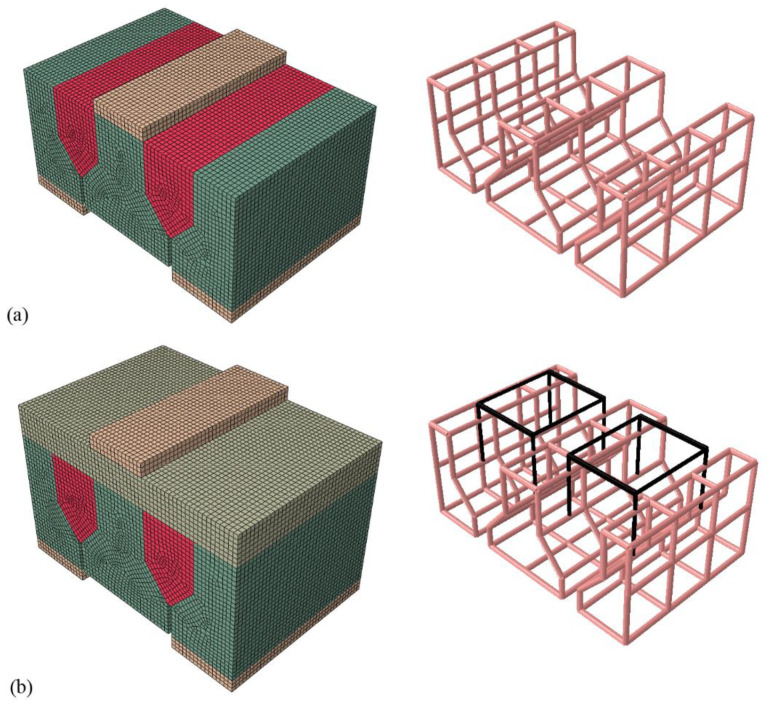
FEMs: (**a**) the SDS test for Type I specimen; (**b**) the SDS test for Type III specimen.

The concrete blocks, joints, overlays, and steel plates were modeled with 8-node brick elements. Steel bars were modeled with 2-node trusses. The concrete damaged plasticity (CDP) model was used to model concrete behavior. The classical metal plasticity model with isotropic hardening was used to model steel behavior. Hard contact and friction were used to model the interface between the specimen and steel plate. The traction–separation constitutive model was used to model the interface of the block and joint. Full bond was used at the overlay–block and overlay–joint interfaces. Steel bars were embedded in the whole model. The two FEMs are shown in Figure 15.

### 4.2. Traction–Separation Constitutive Model

The traction–separation constitutive model offers a method to model thin bonded interfaces whose geometric thickness may be considered to be zero for all practical purposes [52]. The constitutive thickness of interfaces is 1 unit by default and can be specified by users. Note that other input parameters in the model are based on the defined thickness value. The default number 1 was adopted in this study, and the length unit is mm.

The whole traction–separation model contains linear elastic traction–separation, damage initiation criteria, and a damage evolution model. The linear elastic traction–separation model contains stiffness parameters *E_n_*, *E_s_*, and *E_t_*, representing normal and tangential stiffness components. The quadratic nominal stress criterion was used in this study as a damage initiation criterion and can be represented as

(5) $${\left(\frac{\langle t_{n}\rangle }{t_{n}^{0}}\right)}^{2}+{\left(\frac{t_{s}}{t_{s}^{0}}\right)}^{2}+{\left(\frac{t_{t}}{t_{t}^{0}}\right)}^{2}=1$$

where *t_n_*, *t_s_*, and *t_t_* represent the normal and tangential stress components; $t_{n}^{0}$, $t_{s}^{0}$, and $t_{t}^{0}$ represent peak values of the nominal stress when the deformation is either purely normal to the interface or purely in the first or the second shear direction. The symbol <> used in Equation (5) represents the Macaulay bracket with the usual interpretation. Damage is initiated only when the left part of Equation (5) equals 1. Once damage initiation has occurred, damage evaluation is determined on the fracture energy. In this study, the stiffness, peak stress, and fracture energy components in different directions were assumed to be the same [13], referred to as *E*, *t*_0_, and *G* below.

In the FEM, the initial values of stiffness components were set to 380 MPa, deduced from the direct shear test on specimens composed of concretes cast at different times conducted by Harries et al. [50]. They used concretes with 28-day compressive strengths of 41.5 MPa and 29.1 MPa for old and new parts, respectively, and the interfaces were roughened to at least 6.4 mm amplitude before casting the new part. The initial values of peak stress components were determined by *V_c_* divided by the connection area projected to the vertical plane. The initial values of fracture energy components were set to 0.1 N/mm based on that of concrete [53]. Then, these material parameters were calibrated during simulation so that the *V-*∆ curves of the model could match the experimental results.

### 4.3. FE Results

**Figure 16:**
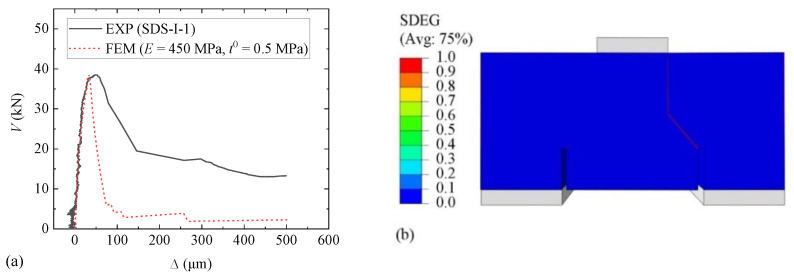
Results for Specimen SDS-I-1: (**a**) *V*-∆ curves; (**b**) damage status.

A comparison of the *V-*∆ curves resulting from the calibrated FEM and the experimental result (EXP) for Specimen SDS-I-1 is shown in Figure 16a. *E* and *t*_0_ were calibrated to 450 MPa and 0.5 MPa, respectively. Values of fracture energy components ranging from 0.01 to 1.00 N/mm had been tried during the simulation, but little changed in the shape of *V-*∆ curves. The curves matched well in the ascending part but not in the descending part. However, the descending part is of minor importance; thus, the result is acceptable. The scalar stiffness degradation (SDEG), indicating the damage degree of model elements, is shown in Figure 16b, where SDEG = 0 indicates intact status and SDEG = 1 failure status. The damage status is similar to that shown in Figure 8a. For all Type I specimens, values of stiffness components with a range of 120 to 450 MPa and peak stress components with a range of 0.5 to 0.6 MPa are recommended.

**Figure 17:**
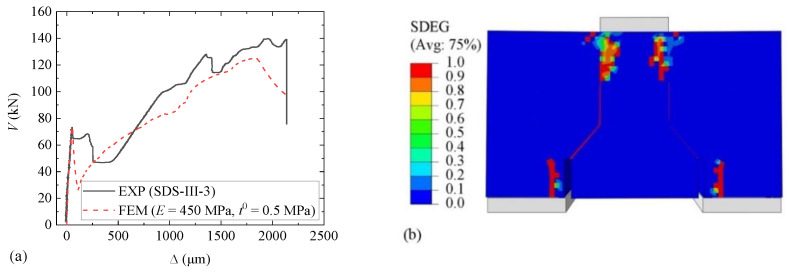
Results for Specimen SDS-III-3: (**a**) *V*-∆ curves; (**b**) damage status.

A comparison of the *V-*∆ curves for the second FEM result using material parameters obtained from the first FEM and the experimental result of SDS-III-1 is shown in Figure 17a. Values of stiffness components and peak stress components were 450 MPa and 0.5 MPa, respectively, the same as those for Specimen SDS-I-1. Both ascending parts before and after cracking showed good agreement, and the damage status shown in Figure 17b is similar to that in Figure 9a.

## 5. Conclusions

Static and dynamic bi-shear tests were conducted on four types of transverse connections used in adjacent box-beam bridges to evaluate their shear transfer performance before and after cracking. FEMs were developed to calibrate and validate the interfacial material parameters. Based on the results obtained from this study, the following conclusions can be drawn:

1. Adding overlays and reinforcing bars increased *V_c_* and *V_u_* by 53% and 235%, respectively, for the Type I specimen, and by 21% and 187%, respectively, for the Type II specimen.
2. All four types of connection could remain intact under the dynamic loading under approximately 0.6*V_c_*. When the Type I and Type II connections cracked, the load transfer failed quickly under cyclic loading. Although the Type III and Type IV connections cracked, the load transfer could still be maintained under a relatively low force or displacement cyclic loading.
3. The *V-*∆ curves for Type I and Type II could be simplified as a bilinear curve; the *V-*∆ curves for Type III and Type IV could be simplified as a combination of two bilinear curves corresponding to before and after cracking performance, respectively. The corresponding formulas, Equations (3) and (4), were proposed for engineering applications.
4. FEM results agreed well with EXP results. Values ranging from 120 MPa to 450 MPa for stiffness components and values ranging from 0.5 MPa to 0.6 MPa for peak stress components were recommended for interface materials with a unit thickness (1 mm) when using the traction–separation model.

## Figures and Tables

**Table 1:** Mix design for C30.

Constituent	C30 (kg/m^3^)	C40 (kg/m^3^)
Aggregate	973	1000
Sand	845	791
Cement type 42.5/52.5 ^1^	284	300
Water	92	89
Fly ash	35	20
Mineral powder	63	94
Polycarboxylates high-performance water-reducing admixture	8	8

^1^ Cement type 42.5 was used for C30, and cement type 52.5 was used for C40.

**Table 2:** Results of SDS test.

Specimen	*V_c_* (kN)	*V_p_* (kN)	*V_d_* (kN)	*V_u_* (kN)	*k_c_* (kN/μm)	*k_p_* (kN/μm)	∆*_p_* (μm)	∆*_u_* (μm)	*C_o_* (μm)
SDS-I-1	38.5	38.5		38.5	1.61		50	50	
SDS-I-2	49.0	49.0		49.0	0.87		72	72	
SDS-I-3	43.8	43.8		43.8	0.98		78	78	
CFS-I-1 ^1^	45.4	45.4		45.4	1.15		55	55	
Average	44.2	44.2		44.2					
SDS-II-1	39.3	43.0		43.0	1.52		76	76	
SDS-II-2	53.2	55.5		55.5	0.43		226	226	
SDS-II-3	56.4	56.4		56.4	1.31		57	57	
CFS-II-2 ^1^	72.2	72.2		72.2	1.60		45	45	
Average	54.8	56.0		56.0					
SDS-III-1 ^2^	62.4	62.4	28.7	-		-	27	-	-
SDS-III-2	62.7	62.7	18.8	160.1	1.81	0.04	40	4020	4378
SDS-III-3	71.7	71.7	46.9	139.1	1.95	0.05	68	2132	3524
CFS-III-1 ^1^	73.0	73.0	53.2	145.1	2.01	0.04	55	2864	4205
Average	67.5	67.5		148.1					
SDS-IV-1	64.0	64.0	22.4	126.1	1.93	0.04	63	3337	4743
SDS-IV-2	58.0	60.5	32.0	151.6	1.04	0.04	202	3287	4502
SDS-IV-3	55.3	55.3	27.3	164.1	2.80	0.03	55	5289	6389
CFS-IV-1 ^1^	80.0	80.0	30.9	172.5	2.40	0.04	36	4092	7560
CFS-IV-2 ^1^	78.7	78.7	29.2	155.3	2.21	0.04	45	4080	5020
Average	66.2	70.0		160.9					

^1^ The specimen was subjected to SDS test after CFS test. ^2^ The test was terminated when the load dropped for the first time.

**Table 3:** Results of CFS test.

Specimen	*V* (kN)	*V*/*V_c_* (%)	Cracked?	Failed?	Number of Cycles (Count)
CFS-I-1	30	68	No	No	1400
CFS-I-2	35	79	Yes	Yes	10
CFS-I-3	35	79	Yes	Yes	121
CFS-II-1	35	64	No	No	1400
CFS-II-2	40	73	Yes	Yes	1314
CFS-II-3	40	73	No	No	1400
CFS-III-1	40	59	No	No	1400
CFS-III-2	45	67	Yes	No	28
CFS-III-3	45	67	Yes	No	20
CFS-IV-1	40	60	No	No	1400
CFS-IV-2	45	68	No	No	1400
CFS-IV-3	50	76	Yes	No	25

**Table 4:** Results of CDS test.

Specimen	$V_{c}^{d}\;(\mathrm{kN})$	$V_{u}^{d}\;(\mathrm{kN})$	Failed?
CFS-I-1	38.4		Yes
CFS-I-2	48.5		Yes
CFS-I-3	44.9		Yes
Average	43.9		
CFS-II-1	55.3		Yes
CFS-II-2	44.2		Yes
CFS-II-3	55.8		Yes
Average	51.8		
CFS-III-1	61.7	130.6	Yes
CFS-III-2	66.4	147.9	Yes
CFS-III-3 ^1^	-	-	
Average	64.1	139.3	
CFS-IV-1 ^2^	62.4	>93.5	No
CFS-IV-2 ^2^	73.4	>125.0	No
CFS-IV-3	72.0	127.1	Yes
Average	69.3	-	

^1^ The specimen was damaged due to improper operation. ^2^ The preset displacement was small and the specimen did not fail.

## Data Availability

Data are contained within the article.
